# Bioinformatics research at SBB-2019

**DOI:** 10.1186/s12859-020-03712-1

**Published:** 2020-09-14

**Authors:** Yuriy L. Orlov, Elvira R. Galieva, Tatiana V. Tatarinova

**Affiliations:** 1 The Digital Health Institute, I.M.Sechenov First Moscow State Medical University (Sechenov University), 119991 Moscow, Russia; 2 Novosibirsk State University, 630090 Novosibirsk, Russia; 3 Agrarian and Technological Institute, Peoples’ Friendship University of Russia (RUDN), 117198 Moscow, Russia; 4 La Verne University, La Verne, CA 91750 USA; 5 Department of Fundamental Biology and Biotechnology, Siberian Federal University, 660074 Krasnoyarsk, Russia; 6 Vavilov Instutute of General Genetics RAS, 119991 Moscow, Russia

This Special Issue of BMC Bioinformatics “Systems Biology and Bioinformatics” collates the papers presented at the 11th Young Scientists School “Systems Biology and Bioinformatics”-2019 (SBB-2019) hold in summer 2019 in Novosibirsk, Russia (http://conf.bionet.nsc.ru/sbb2019/en/). The issue contains material on classical sequence analysis, bioinformatics applications in medicine, and the theoretical research on gene network structure and dynamics. This traditional school on bioinformatics is organized annually since 2008 by the Institute of Cytology and Genetics of the Siberian Branch of the Russian Academy of Sciences and Novosibirsk State University. In 2019 we had several large international meetings in Russia - in Novosibirsk and Moscow presenting materials on genetics and bioinformatics. The name of the special issue - SBB-2019 reflects the main topics of the event. Traditionally we select the best conference and school materials to be presented at BMC Genomics (https://bmcgenomics.biomedcentral.com/articles/supplements/volume-21-supplement-7) and BMC Genetics post-conference publications [1–4], see also (https://bmcbioinformatics.biomedcentral.com/articles/supplements/volume-20-supplement-1).

The SBB Schools in Novosibirsk are satellite events for BGRS\SB multiconference organized biannually. In 2019, the SBB-2019 School was held as a broad-scope independent meeting with science and education components. Other Special Issues in the fields of genomics, bioinformatics, microbiology, and medical genomics accompany this Special Issue in bioinformatics, published as a part of the following series: BMC Genomics and BMC Medical Genomics, BMC Genetics and BMC Medical Genetics, as well as in BMC Microbiology. In 2018, the conference highlights were organized into the Special Issues with reports from the BGRS\SB-2018 conference and earlier from Belyaev Readings-2017 (http://conf.bionet.nsc.ru/belyaev100/en) [5, 6]. We continued the BMC Bioinformatics special issues in 2019 [7, 8]. At the time of this paper writing, the BGRS\SB-2020 event passed in Novosibirsk (https://bgrssb.icgbio.ru/2020/). For the first time, it was in an online format. We believe such events and public discussion at the platforms of international publishers bring attention of the readers [8, 9] (https://www.frontiersin.org/research-topics/14266/bioinformatics-of-genome-regulation; https://peerj.com/collections/72-bgrs-sb-2020/).

The papers comprising this issue of BMC Bioinformatics were discussed at the SBB-2019 School in Novosibirsk. We open up this Special Issue by classical work on protein sequence analysis by Valery Polyanovsky and colleagues [10] (this issue). The quality of sequence alignment is determined by the substitution matrix and parameters of the insertion-deletion penalty function. The authors conducted a numerical experiment using a representative sample of existing matrices of various types and origins, such as the classic evolutionary matrix series (PAM, Blosum), structural alignment based matrices, and contact energy matrix. Hence, they made an optimal choice of the substitution matrix and the penalty parameters. The best alignment quality is achieved with matrices corresponding to the most substantial evolutionary distance: Gonnet, VTML250, PAM250, MIQS, and Pfasum050. The same property is inherent in matrices not only of evolutionary origin but also of another background corresponding to a significant evolutionary distance. Therefore, matrices based on structural data show alignment quality close enough to its value for evolutionary matrices — this strategy agrees with the idea that the spatial structure is more conservative than the protein sequence. The study by Anastasia Anashkina et al. [11] (this issue) continues the protein structure analysis topic. The authors consider S-glutathionylation - the formation of disulfide bonds between the tripeptide glutathione and cysteine residues of the protein, protecting them from irreversible oxidation [12]. Based on the heptapeptide sequences, a position-specific matrix was created by analyzing the protein sequence near the cysteine residue. The authors proposed an effective method for calculating the glutathionylation propensity score, which utilizes the position-specific matrix and a criterion for predicting glutathionylated peptides.

The review by Mila Efimenko and colleagues [13] (this issue) is in the medical bioinformatics field. The authors discuss medical image recognition technologies to detect melanomas using neural networks. They searched the PubMed database for systematic reviews and original research papers. The authors considered convolutional and deep-learning neural networks as well as the fuzzy clustering or World Cup Optimization algorithms in analyzing dermatoscopic images. They have shown that neural networks show higher specificity, accuracy, and sensitivity than dermatologists to evaluate the disease features.

Timofey Ivanisenko and co-authors [14] (this issue) present a new module of ANDSystem (Associative Network Discovery System) for the search of knowledge in the scientific literature. This application extends the functionality of the popular ANDSystem tool for automatic text mining of scientific publications [15, 16]. Currently, there are several commercial automated services allowing users to reconstruct molecular-genetic networks using the data automatically extracted from the texts of scientific publications, such as STRING, Pathway Commons, MetaCore, and Ingenuity. The presented ANDDigest system is a new web-based module of the ANDSystem, permitting searching within PubMed by using dictionaries from the ANDSystem tool and sets of user-defined keywords. The popular search engines, such as Google Scholar, PubMed, and Scopus, are powerful universal tools for keyword-based document searches without linking to any specific field of knowledge. Text-mining of scientific publications in bioinformatics by the proposed system is an alternative to such lookup, providing automated extraction and formalized representation of accurate biomedical information [17, 18].

Dr. Likhoshvai co-authored two manuscripts on theoretical investigations of gene networks. Sadly, Dr. Likhoshvai has recently passed away, leaving several unpublished works to be completed by his colleagues. The publications were prepared by Vladimir P. Golubyatnikov [19], and Tamara M. Khlebodarova [20] (this issue). They discuss the dynamic behavior of gene networks and oscillating models. The regulatory feedback loops that present in the structural and functional organization of molecular-genetic systems and the phenomenon of the regulatory signal delay, a period between the moment of signal reception and its implementation, provide natural conditions for complicated dynamic regimes in these systems. In [19], the authors studied the dynamical properties of models of simplest circular gene networks regulated by negative feedback mechanisms. They have demonstrated the existence and stability of oscillating trajectories (cycles) in these models. From the evolutionary viewpoint, the configuration of loops with negative feedback regulation is a more favorable mode for cell functioning. It can be accomplished by a simple blockage of the transcription initiation site by the regulatory protein without the formation of complex structures that interact with the RNA polymerase. This type of regulation is widespread, and its simplest example is a sub-system containing just one gene, which controls its expression by a negative feedback mechanism, representing the minimal regulatory circuit, acting on itself. The system of differential equations presents the simplest model of artificial molecular triggers [21].

We note previous publications on gene network models by the authors, presented at “Systems biology and bioinformatics” Schools in Novosibirsk, Russia [22, 23], the publication on chaos in gene expression dynamics [24–26]. The previous works by V.A.Likhoshvai were presented at Systems Biology and Bioinformatics (SBB) Schools since 2012 [24] and discussed in [27, 28].

Tamara Khlebodarova and colleagues applied gene network models for the analysis of dynamic regulation of synaptic protein that may cause neuropsychiatric diseases [20] (this issue). Fragile X Mental Retardation Protein, which regulates the efficiency of dendritic mRNA translation in response to the stimulation of metabotropic glutamate receptors at excitatory synapses of the hippocampal pyramidal cells [29]. Its activity is regulated via positive and negative regulatory loops that function in different time ranges, which is an absolute factor for the formation of chaotic regimes that lead to disrupted proteome stability. A mathematical model that describes the maintenance of a specific pool of active receptors on the postsynaptic membrane via two mechanisms – de novo synthesis of receptor proteins and restoration of protein function during the recycling process – has been developed. A similar analysis of the dynamic behavior of gene networks was applied to local translation at activated synapses, also related to mental disorders [30]. The results on gene model simulation suggested that the chaotic behavior of the network parameters is quite common [31–33]. Note the publication by Dr. Likhoshvai on this topic in BioMed Central journals by the SBB School materials in 2014 (https://bmcmicrobiol.biomedcentral.com/articles/supplements/volume-16-supplement-1;https://bmcgenomics.biomedcentral.com/articles/supplements/volume-15-supplement-12). The original work on the dynamic of cell replications [33] continued in recent work [34] in 2019. His work on gene network modeling served as background on current applications by his disciples in databases [26, 35], plant science models [36].

Therefore, this issue includes reports of recent bioinformatics applications in protein sequence and structure analysis, text mining, gene networks modeling. We aim to support international exchange and education in new forms via the schools and competitions for young scientists (http://conf.bionet.nsc.ru/sbb2019/en/). Next, SBB-2020 School in Russia is scheduled for September 2020. BGRS\SB-2020 (Bioinformatics of Genome Regulation and Structure \ Systems Biology) multiconference just over in July 2020 holding series of symposia and workshops (https://bgrssb.icgbio.ru/2020/), and following journal publications (https://www.mdpi.com/journal/ijms/special_issues/Bioinformatics_Genomics; https://peerj.com/collections/72-bgrs-sb-2020/). We invite our readers worldwide to attend our next events on Systems Biology and Bioinformatics in Russia, as well as the systems biology meetings in Moscow - Digital Medicine Forum (https://forum.digital/).
